# Non-invasive nitric oxide use for pulmonary hypertension and pulmonary vascular disease associated with bronchopulmonary dysplasia in preterm infants

**DOI:** 10.1038/s41372-026-02687-w

**Published:** 2026-04-15

**Authors:** Aastha Chandra, Danielle R. Rios, Daniel Dagle, Patrick J. McNamara, Adrianne R. Bischoff

**Affiliations:** 1 Carver College of Medicine, University of Iowa, Iowa City, IA USA; 2 Division of Neonatology, Department of Pediatrics, University of Iowa, Iowa City, IA USA; 3 Interdisciplinary Graduate Program in Informatics, University of Iowa, Iowa City, IA USA; 4 Department of Internal Medicine, University of Iowa, Iowa City, IA USA

**Keywords:** Paediatrics, Hypertension, Vascular diseases

## Introduction

Inhaled nitric oxide (iNO) is a pulmonary vasodilator used via invasive ventilation in the treatment of neonates with hypoxemic respiratory failure (HRF) greater than 34 weeks’ gestational age. In extremely preterm neonates with echo-confirmed pulmonary hypertension (PH), >50% have oxygenation improvement following iNO administration even though its use remains controversial [1]. Use of iNO in preterm infants with bronchopulmonary dysplasia (BPD) associated PH and pulmonary vascular disease (PVD) via non-invasive ventilator devices has increased. Our objective was to investigate clinical and echocardiography response of non-invasive iNO in preterm infants with PVD and/or BPD-associated PH.

## Methods

A retrospective cohort study (July 2019–February 2024) of patients born at <30 weeks gestational age who received iNO via non-invasive devices, including nasopharyngeal tube (NP) or RAM cannula® to administer continuous positive airway pressure (CPAP) was conducted. Infants were identified through the Neonatal Hemodynamics database by records of non-invasive iNO administration and cross-referenced with the medical charts. Inhaled nitric oxide was administered at the discretion of the treating neonatologist following a targeted neonatal echocardiography (TNE) diagnosis of PVD and/or BPD-associated PH in the setting of escalating oxygen requirement or concern for pulmonary vascular reactivity despite optimization of lung recruitment. PVD was defined as pulmonary vascular index (PVRi = right ventricular ejection time indexed by pulmonary artery acceleration time) ≥4 and/or presence of notched pulmonary artery Doppler. PH was defined by right ventricular systolic pressure (RVSp) ≥ 30 mm Hg from a tricuspid regurgitant jet and/or end-systolic eccentricity index (sEI) ≥ 1.3. Pre-iNO TNE was obtained up to 48 h prior to iNO initiation and post-iNO TNE was recorded within 7 days of initiation (while still receiving iNO). The primary outcome was echocardiography response to iNO, defined as resolution of PH (RVSp <30 mmHg and/or sEI < 1.3) or PVD (PVRi <4 without notching). Secondary outcomes included changes in sEI, PVRi, ejection fraction, tricuspid annular plane systolic excursion, RV fractional area change, RVs’ velocity, and left and right ventricular outputs. Inhaled nitric oxide was initiated at 20 ppm in most cases and weaned gradually based on serial TNE findings, oxygen requirement, and clinical stability. Weaning was typically performed in decrements of 5 ppm until a dose of 5 ppm and then in decrements of 1 ppm to off. Weaning was typically done once echocardiographic markers of PH/PVD improved and fraction of inspired oxygen remained stable or improved. In TNE non-responders, iNO was weaned as per discretion of the attending neonatologist.

In our unit, the presence of a hemodynamically significant patent ductus arteriosus or atrial-level shunt was evaluated prior to initiation of iNO. Infants with large left-to-right shunts contributing to pulmonary overcirculation or significant left ventricular systolic dysfunction (typically defined as an ejection fraction <50%) were not considered ideal candidates for isolated pulmonary vasodilator therapy and were managed according to the underlying pathophysiology. Infants with significant systemic hypertension and multiparametric TNE findings concerning left ventricular diastolic impairment were also not treated with iNO.

Non-invasive iNO was typically considered in older preterm infants who were no longer intubated but had established BPD and echocardiographic evidence of PVD/PH despite optimization of respiratory support. In this setting, our practice typically favors iNO as first-line therapy over systemic or oral agents due to its selective pulmonary vasodilator effects, rapid onset of action, and utility in assessing cyclic guanosine monophosphate pathway responsiveness, while potentially minimizing ventilation-perfusion mismatch in heterogeneous lung disease.

At our center, we use standardized oxygen saturation targets (SpO_2_) at ≥97% to modify the risk of retinopathy of prematurity (ROP) in infants with a diagnosis of pre-threshold ROP (Zone I Stage 1 or 2, Zone II Stage 2 or 3) [2]. Since this practice minimizes the likelihood of significant changes in FiO_2_, we analyzed SpO_2_ averages for 1 h before pre- and post-iNO TNE and 6 h following iNO administration.

The study was approved by the Institutional Review Board at the University of Iowa with waiver of informed consent.

## Results

Twelve patients were identified with a mean ± SD gestational age of 24.8 ± 1.67 weeks and birth weight of 550 ± 153 g. Nine (75%) patients met diagnostic criteria for PH, while 3 (25%) had PVD exclusively without PH at the time of iNO initiation. At the time of iNO initiation, infants were supported on NP-CPAP, NP neurally adjusted ventilator assist or RAM CPAP. Capillary blood gases within the day of initiation demonstrated a mean ± SD pH of 7.39 ± 0.02 and a carbon dioxide level of 57 ± 7 mmHg, without significant evidence of acute hypercapnic respiratory failure. At the time of iNO initiation, five infants (four non-responders and one responder) were already receiving other pulmonary vasodilator agents (one infant was receiving sildenafil, three infants were receiving inhaled epoprostenol and one infant was receiving both sildenafil and inhaled epoprostenol). During the therapy with non-invasive iNO, two infants were started on other pulmonary vasodilators within 24 h and one additional infant was started after 24 h. Additionally, five infants were discharged from the neonatal intensive care unit while on pulmonary vasodilators (four on sildenafil and one on riociguat). A starting and maximum dose of 20 ppm was used in 9 (75%) and 8 (66.7%), respectively. Duration of therapy ranged from 189 to 947 h with a mean of 462 ± 244 h. Of the 9 infants with PH, 3 (33%) demonstrated echocardiographic response, whereas 2 out of 3 (67%) infants with isolated PVD without PH met response criteria. Table 1 summarizes the clinical and echocardiography characteristics of responders versus non-responders. Among responders, mean sEI decreased by 12.4% and mean PVRi decreased by 13.2% following iNO initiation, whereas non-responders demonstrated minimal change (1.5% and 4.2% increase, respectively) (Supplementary Fig. 1). Responders had indices suggesting higher degree of lung disease, including a higher rate of NP-NAVA versus RAM Cannula and a trend towards higher PEEP and modified RSS. Other baseline clinical or TNE characteristics were similar between groups. Although echocardiographic responders demonstrated improvement in TNE PH/PVD indices, oxygen saturation did not significantly change pre/and post-iNO (*p* = 0.191), likely reflecting institutional saturation targets for pre-threshold ROP and limited opportunity for reduction in fraction of inspired oxygen.

**Table 1:** Clinical and echocardiography characteristics according to responsiveness to inhaled nitric oxide.

	Responders (*n* = 5)	Non-responders (*n* = 7)	*p*
Male (%)	2 (40)	2 (28.6)	1
Gestational age (weeks)	23.3 [23, 27.3]	24.8 [23.8, 26.3]	0.876
Birth weight (grams)	505 [447, 780]	560 [390, 580]	0.755
Age at iNO start (days)	79 ± 114	123 ± 34	0.815
Postmenstrual age at iNO start (weeks)	35 ± 6	42 ± 5	0.061
Weight at iNO start (grams)	3061 ± 1858	3225 ± 1115	0.852
Ventilation prior to starting iNO (%)			0.030
- RAM CPAP	1 (20)	6 (85.7)	
- NP-CPAP	1 (20)	0	
- NIV-NAVA	3 (60)	1 (14.3)	
Pre-iNO PEEP (cmH20)	11 ± 2	8 ± 2	0.072
Pre-iNO FiO_2_*100 (%)	67 ± 19	53 ± 16	0.200
Pre-iNO modified RSS (PEEP*FiO2)	6.3 ± 1.3	3.6 ± 2.2	0.064
Starting dose of iNO (%)			0.424
- 20 ppm	3 (60)	6 (85.7)	
- 10 ppm	1 (20)	0	
- 5 pmm	1 (20)	1 (14.3)	
Duration of iNO therapy (h)	441 ± 283	477 ± 235	0.817
Pre-iNO Echo			
Time of Echo in relationship to iNO start (h)	16 ± 25	11 ± 8	0.074
Diagnosis of PH	3 (60)	6 (85.7)	1
Diagnosis of PVD	3 (60)	6 (85.7)	1
RVSp (mmHg)	50 ± 11	49 ± 8	0.878
End-systolic eccentricity index	1.25 ± 0.2	1.25 ± 0.3	0.972
Pulmonary vascular resistance index	4 ± 0.8	5.1 ± 1.1	0.108
Ejection fraction by Simpson's biplane (%)	70 ± 6	65 ± 3	0.053
Tricuspid annular plane systolic excursion (mm)	10.4 ± 3.3	11.8 ± 3.7	0.499
RV fractional area change	0.47 [0.4, 0.47]	0.41 [0.38–0.44]	0.517
RV s’ from tissue doppler imaging (m/s)	7.8 ± 1	7.3 ± 1.2	0.495
Left ventricular output (mL/kg/min)	197 ± 47	201 ± 47	0.903
Right ventricular output (mL/kg/min)	191 ± 59	185 ± 32	0.418
Right ventricular ejection time (ms)	199 ± 16	188 ± 25	0.377
Pulmonary artery acceleration time (ms)	51 ± 14	41 ± 4	0.097

*iNO* inhaled nitric oxide, *NP* nasopharyngeal, *CPAP* continuous positive airway pressure, *NAVA* neurally adjusted ventilatory assist, *PEEP* positive end-expiratory pressure, *FiO*_2_ fraction of inspired oxygen, *RSS* respiratory severity score, *PH* pulmonary hypertension, *PVD* pulmonary vascular disease, *RV* right ventricular.

A total of five infants were discharged from the neonatal intensive care unit while on pulmonary vasodilators (four on sildenafil and one on riociguat). A total of four out of five of these infants were considered non-responders to non-invasive iNO. There were two infants who underwent tracheostomy during their admission and all babies survived to discharge.

## Discussion

In this retrospective study, we demonstrate that a proportion of preterm infants with TNE-confirmed PVD and/or BPD-associated PH respond to non-invasively delivered nitric oxide. Nearly half of the cohort demonstrated echocardiography improvement post-iNO. These patients were on different modes of respiratory support, with responders more frequently supported on NP-CPAP or NIV-NAVA, while non-responders were almost exclusively on RAM cannula. This may suggest iNO delivery may vary between noninvasive methods. It is also notable that responders overall demonstrated a trend toward higher PEEP and RSS, suggesting potentially greater respiratory support requirements, although these differences did not reach statistical significance. The higher proportion of responders among infants with isolated PVD compared to those with established PH may suggest that earlier or less advanced PVD is more amenable to selective pulmonary vasodilation. Conversely, infants with established PH may have more fixed pulmonary vascular remodeling or greater contribution from parenchymal lung disease, limiting acute responsiveness.

These findings align with prior studies of non-invasive iNO use in neonates with HRF and congenital diaphragmatic hernia (CDH) [3, 4]. iNO delivery in patients with HRF was associated with improved oxygenation while patients were transitioned from invasive ventilation to bubble CPAP, augmenting airway recruitment [3]. In patients with CDH, who often experience a late or protracted course of PH, iNO delivery via nasal cannula demonstrated utility in improving SpO_2_ while reducing exposure duration to mechanical ventilation [4]. Differences in response across non-invasive interfaces raise the possibility that delivered iNO concentration at the nasopharyngeal level may vary by device and flow characteristics. These may reflect factors beyond lung disease severity, including variability in delivered nitric oxide concentration and interface leak. Stability of mean airway pressure and patient-device synchrony. These variables may influence both effective pulmonary vasodilator delivery and alveolar recruitment. Future studies evaluating actual delivered concentration and titration strategies across non-invasive ventilatory modalities may optimize therapeutic response and represent an important area for investigation.

Overall, these data together suggest that non-invasive iNO may reduce intrapulmonary shunting in select patients by improving ventilation and redistributing blood flow to previously unrecruited alveoli. The study was limited by its retrospective design, small sample size, and lack of standardized timing for post-iNO TNE. Effectiveness was determined by echocardiographic indices without direct measurements of delivery iNO concentration across non-invasive interfaces, and formal intra- and inter-observer variability analysis was not performed. In addition, the absence of a control group and the concurrent use of other pulmonary vasodilators during therapy limit causal inference. Larger prospective studies are needed to better stratify outcomes by non-invasive delivery modality and to more rigorously define the subset of preterm infants with TNE-confirmed PVD or BPD-associated PH who may benefit from this strategy.

## Supplementary information

Supplemental Figure 1
