# Supplementary material for: Non-invasive nitric oxide use for pulmonary hypertension and pulmonary vascular disease associated with bronchopulmonary dysplasia in preterm infants
Source: J Perinatol. 2026 Apr 15;46(7):1334–6. doi: 10.1038/s41372-026-02687-w (PMC13423795; doi:10.1038/s41372-026-02687-w)
Supplement: Supplementary file 1 — Supplemental Figure 1 [file 41372_2026_2687_MOESM1_ESM.pdf]

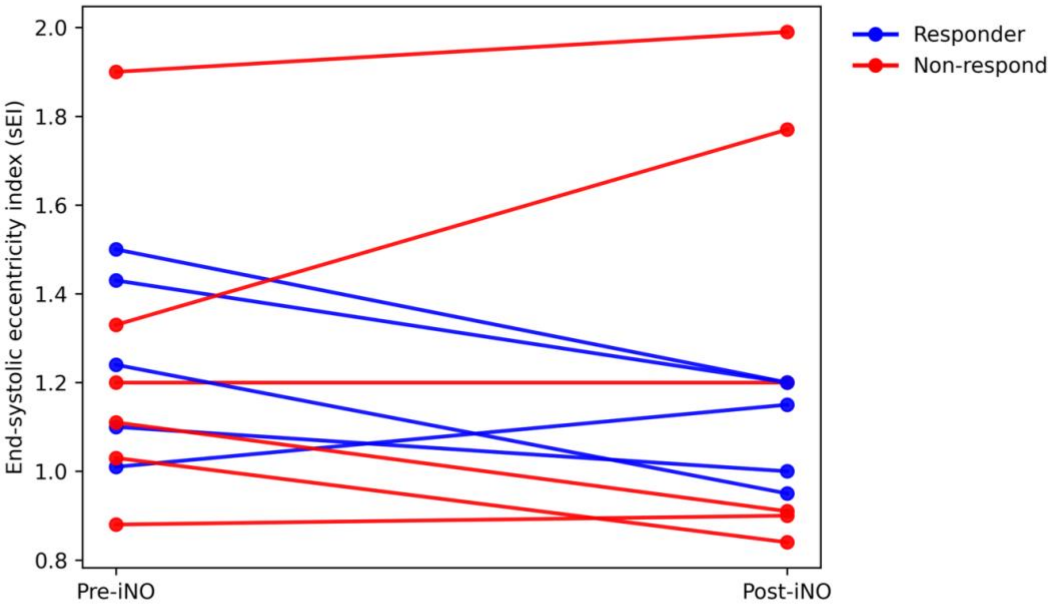

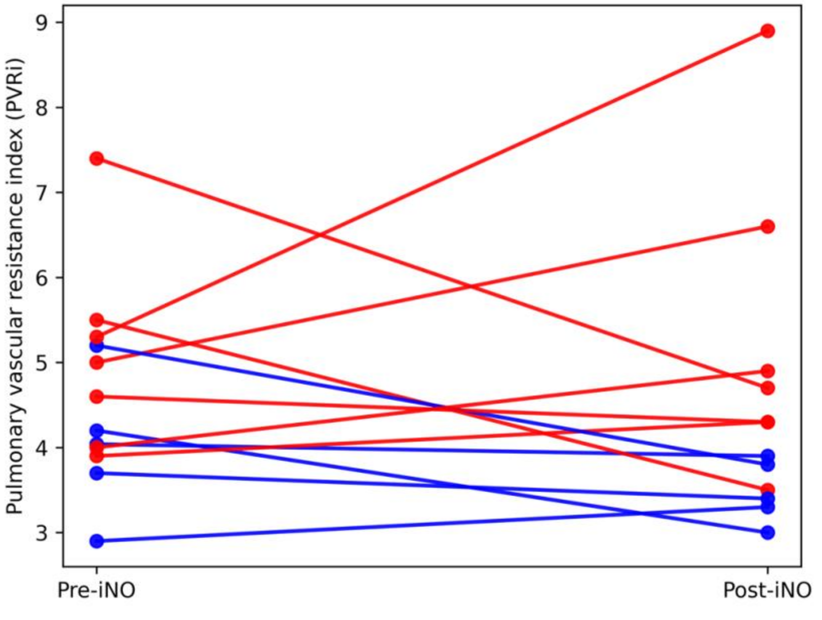

Supplemental Figure 1: Individual patient trajectories of end-systolic eccentricity index (sEI, Panel A) and pulmonary vascular resistance index (PVRi, Panel B) before and after initiation of non-invasive inhaled nitric oxide, with responders shown in blue and non-responders in red.
